# Structure, Properties, and Corrosion Behavior of Ti-Rich TiZrNbTa Medium-Entropy Alloys with β+α″+α′ for Biomedical Application

**DOI:** 10.3390/ma15227953

**Published:** 2022-11-10

**Authors:** Ka-Kin Wong, Hsueh-Chuan Hsu, Shih-Ching Wu, Tun-Li Hung, Wen-Fu Ho

**Affiliations:** 1 Department of Chemical and Materials Engineering, National University of Kaohsiung, Kaohsiung 81148, Taiwan; 2 Department of Dental Technology and Materials Science, Central Taiwan University of Science and Technology, Taichung 40601, Taiwan

**Keywords:** medium-entropy alloy, biomedical alloy, three-phase structure, mechanical properties, elastic modulus, corrosion property

## Abstract

Five Ti-rich β+α″+α′ Ti–Zr–Nb–Ta biomedical medium-entropy alloys with excellent mechanical properties and corrosion resistance were developed by considering thermodynamic parameters and using the valence electron concentration formula. The results of this study demonstrated that the traditional valence electron concentration formula for predicting phases is not entirely applicable to medium-entropy alloys. All solution-treated samples with homogeneous compositions were obtained at a low temperature (900 °C) and within a short period (20 min). All solution-treated samples exhibited low elastic moduli ranging from 49 to 57 GPa, which were significantly lower than those of high-entropy alloys with β phase. Solution-treated Ti_65_–Zr_29_–Nb_3_–Ta_3_ exhibited an ultra-high bending strength (1102 MPa), an elastic recovery angle (>30°), and an ultra-low elastic modulus (49 GPa), which are attributed to its α″ volume fraction as high as more than 60%. The pitting potentials of all samples were higher than 1.8 V, and their corrosion current densities were lower than 10^–5^ A/cm^3^ in artificially simulated body fluid at 37 °C. The surface oxide layers on Ti_65_–Zr_29_–Nb_3_–Ta_3_ comprised TiO_2_, ZrO_2_, Nb_2_O_5_, and Ta_2_O_5_ (as discovered through X-ray photoelectron spectroscopy) and provided the alloy with excellent corrosion and pitting resistance.

## 1. Introduction

High-entropy alloys (HEAs) have four core effects and excellent qualities that are applicable to various fields of engineering [1]. The structures of HEAs and medium-entropy alloys (MEAs) can be calculated and predicted through the calculation of thermodynamic parameters, and some parameters are directly related to the properties of these alloys [2]. For instance, the effect of lattice distortion on mechanical properties can be determined using the difference in atomic radius (δ) value of the alloy, and the mixing enthalpy (ΔH_mix_) value can also be used for evaluating the aggregation or separation between alloying elements [3]. Many scholars have developed biomedical HEAs and MEAs with high mechanical strength, ductility, corrosion resistance, and biocompatibility [3,4,5,6]. However, the elastic moduli of most biomedical HEAs and MEAs are still considerably higher than that of natural human bone (i.e., >30 GPa) [7]. A stress shielding effect may occur around an implant when the elastic modulus of the implant material is considerably greater than that of human bone [8].

In the development of biomedical alloys, metastable beta-titanium (β-Ti) alloys with ultra-low elastic modulus have been favored by numerous researchers [9,10,11]. The molybdenum equivalent ([Mo]_eq_) and valence electron concentration (VEC) methods are commonly used for calculating phase structures in Ti alloy systems. However, the metastable β-Ti alloys with low elastic modulus generally have low strength [9,12]. For instance, the elastic modulus of solution-treated Ti–33Nb–4Sn (36 GPa) was very similar to that of natural human bone, but its yield strength was as low as 107 MPa [9]. Implants with insufficient strength may fracture in the human body, which increases the risk of surgical failure.

A metastable Ti_65_–Zr_18_–Nb_16_–Mo_1_ MEA that combines the design concepts of metastable β-Ti alloys and MEAs was recently developed [13]. Ti_65_–Zr_18_–Nb_16_–Mo_1_ was discovered to exhibit high yield strength (1118 MPa), which was attributable to the contribution of the lattice distortion effect. The yield strength–elastic modulus ratio (×1000) of the alloy was even higher than 18.3. Nevertheless, the elastic modulus of Ti_65_–Zr_18_–Nb_16_–Mo_1_ was still 2 times that of natural human bone (<30 GPa). Conversely, Liu et al. [14] developed a Ti_45_–Zr_37_–Nb_16_–Fe_1_–Mo_1_ MEA with a metastable β phase and yield strength and elastic modulus of 703 MPa and 63 GPa, respectively. After 74–76% cold rolling, the alloy was transformed into a β+α″ phase, and its yield strength and elastic modulus were 1139 MPa and 74 GPa, respectively. Hence, obtaining an elastic modulus similar to that of natural human bone by incorporating metastable β-Ti alloys into MEAs may be difficult.

Regarding biomedical Ti alloys, some research has indicated that dual-phase and three-phase Ti alloys may have a lower elastic modulus than metastable β-Ti alloys [15,16,17]. In an investigation by Tan et al., the elastic moduli of Ti–33Nb–7Zr with β+α″ phase and Ti–23Nb–7Zr with β+α″+α′ phase were found to be as low as 29.0 GPa and 35.9 GPa, respectively, using a nanoindentation test [17]. Furthermore, the phase boundary in dual-phase and three-phase alloys can hinder the slip of a dislocation, improving the strength of the alloys [18]. Although some biomedical β+α″ MEAs were developed through a cold-rolling process [14], no biomedical dual-phase or three-phase HEAs or MEAs with low elastic modulus have been reported. Because of the interaction between the four core effects of HEAs and the dual-phase/three-phase design concept, alloys are expected to be developed that have high strength and low elastic modulus simultaneously.

In this study, five biomedical Ti-rich Ti–Zr–Nb–Ta MEAs with three-phase structure were developed using the thermodynamic empirical formula and VEC method. These MEAs were discovered to have high strength, low elastic modulus, and excellent corrosion resistance. The phase structure, microstructure, mechanical properties, and corrosion resistance of the Ti–Zr–Nb–Ta MEAs were evaluated.

## 2. Materials and Methods

### 2.1. Experimental Procedures

Ti_65_–Zr_33_–Nb_1_–Ta_1_, Ti_65_–Zr_31_–Nb_2_–Ta_2_, Ti_65_–Zr_29_–Nb_3_–Ta_3_, Ti_65_–Zr_27_–Nb_4_–Ta_4_, and Ti_65_–Zr_25_–Nb_5_–Ta_5_ MEAs were fabricated using a commercial vacuum arc-melting and casting system (A-028, DAWNSHINE, Taiwan). In the process, Ti (99.7 wt% pure), Zr (99.9 wt% pure), Nb (99.95 wt% pure), and Ta (99.95 wt% pure) were employed. The oxide layer on all metal sheets was thoroughly removed by using #200 sandpaper before melting. The metal materials for each alloying element were placed in the water-cooled copper crucible in descending order of melting point during melting. Before casting, alloy ingots were remelted and flipped more than six times to achieve constituent-element homogeneity. The dimensions of all the MEA samples were 30 mm × 5 mm × 1 mm. Solution treatment (ST) of each MEA sample was performed using a high-temperature tubular furnace (MTF 12/38/250, Carbolite Gero, Hope, UK) at 900 °C for 20 min under argon atmosphere, and then the sample was quenched in ice water. The surfaces of all MEAs were mechanically polished using colloidal SiO_2_ polishing suspension (0.06 μm) before all analyses were performed, according to ASTM E3 [19]. The samples used for optical microscopy (OM) and electron probe microanalysis (EPMA) were pre-etched in a solution of deionized water, nitric acid, and hydrofluoric acid in a ratio of 80:15:5 (vol.%), according to ASTM E407 [20].

Phase analysis was conducted using X-ray diffraction (XRD; D8-Advance, Bruker, Berlin, Germany) with Cu-Kα radiation at 40 kV, 40 mA, 2θ = 30°–80°, scanning speed = 4°/min, and step size = 0.02°/step. Electron backscatter diffraction (EBSD; SU5000, Hitachi, Tokyo, Japan) was used for grain orientation and phase structure analyses. The microstructures of the alloys were examined through OM (Zeiss, Oberkochen, Germany). Chemical compositions and elemental mapping analyses were performed using scanning electron microscopy (SEM; 6330, JEOL, Tokyo, Japan) with energy-dispersive X-ray spectroscopy (EDS) and EPMA (JXA-8530F, JEOL) with wavelength-dispersive spectroscopy (WDS). The five MEAs’ nominal and measured compositions, obtained through EDS–SEM, are detailed in Table 1. From a clinical practice viewpoint, implants are often subjected to flexural stresses instead of tensile loads during the movement of the human body [21,22]. The mechanical properties of all MEAs were evaluated through a three-point bending test according to ASTM E855 [23], which was performed using a desktop mechanical tester (HT-2102AP, Hung-Ta Instrument, Taichung, Taiwan). The three-point bending experiments were conducted in accordance with the procedure described in a previous study [13].

Potentiodynamic polarization tests were conducted using a potentiostat (PGSTAT12, Autolab, Utrecht, The Netherlands) in phosphate-buffered saline (PBS) at 37 °C and pH 7.4, according to ASTM G5 [24]. The MEA samples, a silver chloride electrode (Ag/AgCl), and a platinum plate were used as the working electrode, reference electrode, and auxiliary electrode, respectively. Before the test, the electrolyte solution was deaerated with nitrogen gas for 30 min. The scan rate and scan range were 0.001 V/s and –0.3 to 1.8 V, respectively, and the tests were begun after 1 h at the open circuit potential to ensure that the PBS solution had stabilized. The PBS solution comprised NaCl (8.0 g/L), KCl (0.2 g/L), Na_2_HPO_4_ (1.44 g/L), and KH_2_PO_4_ (0.24 g/L). The sample and electrolyte solution were stagnant during potentiodynamic polarization tests. The corrosion potential and corrosion current density were obtained using the Tafel curve extrapolation method [25]. In the polarization curve, the intersection of the anodic and cathodic curves is the corrosion potential of the alloy. Extrapolation of the linear portion of the anodic and cathodic curves to the corrosion potential is utilized to determine the corrosion current density. After the potentiodynamic polarization tests, the surface microstructures of the alloys were examined using SEM, and the surface chemical compositions of the alloys were analyzed using X-ray photoelectron spectroscopy (XPS; JAMP-9500F, JEOL) with a monochromatized Al-Kα excitation source (hν = 1486.6 eV) at 10 kV and 10 mA. The XPS binding energies were calibrated by measuring the reference peak of C 1s (binding energies = 284.6 eV).

### 2.2. Thermodynamic Parameter Calculation

The thermodynamic parameters of the Ti_65_–Zr_33_–Nb_1_–Ta_1_, Ti_65_–Zr_31_–Nb_2_–Ta_2_, Ti_65_–Zr_29_–Nb_3_–Ta_3_, Ti_65_–Zr_27_–Nb_4_–Ta_4_, and Ti_65_–Zr_25_–Nb_5_–Ta_5_ MEAs are detailed in Table 2. All the thermodynamic parameters were calculated as follows [2]: ${\Delta S}_{\mathrm{mix}}=R\ln N$, where ΔS_mix_ is the mixing entropy, R is the ideal gas constant (8.3145 J·K^–1^·mol^–1^), and *N* is the number of component elements; ${\Delta H}_{\mathrm{mix}}=4\sum_{i=1,i\neq j}^{n}{\Delta H}_{\mathrm{mix}}^{\mathrm{AB}}c_{i}c_{j}$, where ΔH_mix_ is the mixing enthalpy (–22 ≤ ΔH_mix_ ≤ 7 kJ/mol) and ${\Delta H}_{\mathrm{mix}}^{\mathrm{AB}}$ is the mixing enthalpy of the binary A–B system; $\delta =\sqrt{\sum_{i=1}^{n}c_{i}{\left(1–r_{i}/\bar{r}\right)}^{2}}$, where δ is the difference in atomic radius (0 ≤ δ ≤ 8.5) and c_i_ and r_i_ are the atomic percentage and atomic radius of element i, respectively; $\Omega =\frac{T_{L}{\Delta S}_{\mathrm{mix}}}{\left|{\Delta H}_{\mathrm{mix}}\right|}$, where T_L_ and ΔS_mix_ are the melting point and mixing entropy of the alloy, respectively; and $\mathrm{VEC}=\sum_{i=1}^{n}c_{i}{\left(\mathrm{VEC}\right)}_{i}$, where VEC and (VEC)_i_ are the valence electron concentration of the alloy and the VEC of element i, respectively. All the Greek letters and symbols used in the study are listed in Table 3.

## 3. Results and Discussion

### 3.1. Phase Identification

#### 3.1.1. X-ray Diffraction Analysis

**Figure 1:**
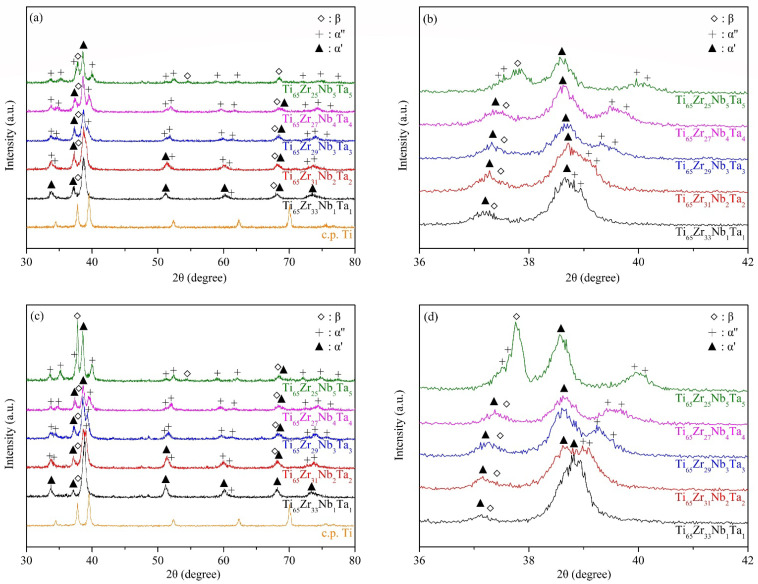
X-ray diffraction patterns of the Ti–Zr–Nb–Ta medium-entropy alloys (MEAs). (**a**) As cast (30–80°), (**b**) as cast (36–42°), (**c**) after solution treatment (ST) (30–80°), and (**d**) after ST (36–42°).

The XRD patterns of the five Ti–Zr–Nb–Ta MEAs under as-cast and ST conditions are presented in Figure 1a–d. All MEAs had a three-phase β+α″+α′ structure before and after ST. In Ti alloy systems, however, when the VEC is < 4.2, the alloy should be the pure HCP (α′) phase [26]. Yuan et al. [27] reported that in Ti/Zr-rich HEAs, when the VEC is < 4.09, the alloy must be the pure HCP (α′) phase. The results obtained in this study thus differ from the predictions made elsewhere [26,27]; the VECs of the five MEAs were all lower than 4.1, but the β and α″ phases were retained. This indicates that under the influence of the four core effects of HEAs, the applicable phase prediction formulas in different alloy systems or compositions may vary considerably. For example, the high-entropy effect promotes the formation of a simple and stable phase structure of the alloy [1] rather than a metastable state, and this eventually leads to a deviation of the alloy’s phase structure from the predicted results. Additionally, the severe composition segregation derived from the characteristics of the multielement composition of HEAs and MEAs may be another factor affecting the phase structure.

**Figure 2:**
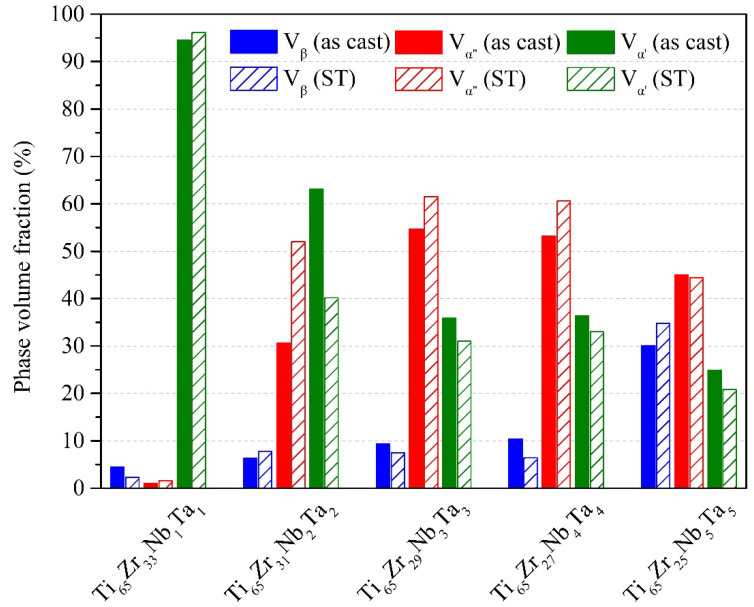
Phase volume fractions (%) of the Ti–Zr–Nb–Ta MEAs under various conditions.

The phase volume fractions of the five β+α″+α′ MEAs before and after ST are listed in Figure 2. Ti_65_–Zr_33_–Nb_1_–Ta_1_ had the greatest α′-phase volume fraction because it had the lowest VEC (4.02). Moreover, the volume fraction of the β phase in Ti_65_–Zr_25_–Nb_5_–Ta_5_ with the highest VEC (4.1) was considerably higher than that of the other four MEAs. Ti_65_–Zr_29_–Nb_3_–Ta_3_, with a VEC of 4.06, exhibited the highest α″-phase volume fraction. Conversely, the volume fractions of the β, α″, and α′ phases in the MEAs were considerably different after ST. The volume fraction of the α′ phase of Ti_65_–Zr_33_–Nb_1_–Ta_1_ was slightly higher after ST. The volume fractions of the α″ phase of Ti_65_–Zr_31_–Nb_2_–Ta_2_, Ti_65_–Zr_29_–Nb_3_–Ta_3_, and Ti_65_–Zr_27_–Nb_4_–Ta_4_ were all considerably higher after ST. The β-phase volume fraction of Ti_65_–Zr_25_–Nb_5_–Ta_5_ was slightly higher after ST. Therefore, the phase structures of the alloys were affected by the segregation of alloy components, which ultimately affected the properties of the alloys.

All of the α′ diffraction peaks for the five MEAs were shifted to low diffraction angles relative to those of Ti (Figure 1a,c), which was attributable to an interlattice distance increase because of the high δ value of the alloy. Furthermore, the enlarged images of the 36–42° region (Figure 1b,d) indicate that the diffraction peaks corresponding to β, α″, and α′ were all shifted when the composition of the alloys was changed. The peaks corresponding to both β and α″ were shifted to high angles when there was more Nb and Ta because the interlattice distances of the β and α″ phases were reduced by the decrease in δ [28,29]. The diffraction peaks corresponding to α′ became separated as the alloy δ value was increased. As the amount of distortion of the cubic unit cell increased, a diffraction peak in the XRD pattern split into widely separated lines [30]. Moreover, in the region 58–63° (Figure 1a,c), the diffraction peaks corresponding to α″ gradually separated with an increase in the VEC. The α″ phase decomposed into an α″ phase with poor β-stabilizing elements and another α″ phase with rich β-stabilizing elements before transformation to β. The α″ phase with poor β-stabilizing elements had a different lattice constant than the α″ phase with rich β-stabilizing elements [31,32].

#### 3.1.2. Electron Backscatter Diffraction Analysis

**Figure 3:**
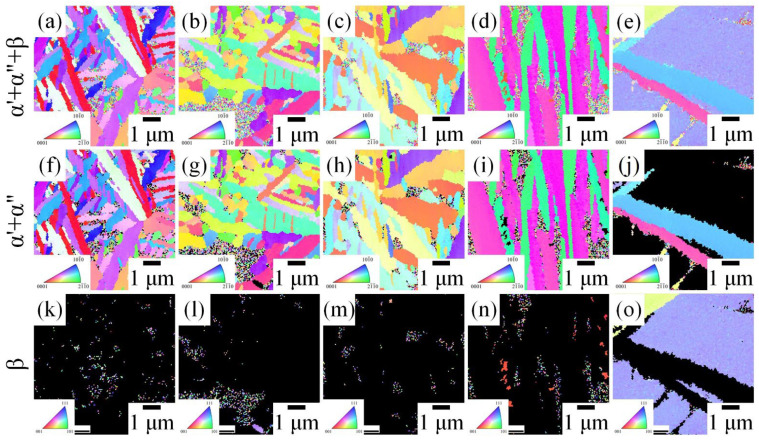
Electron backscatter diffraction images (inverse pole figure and phase map) of the MEAs after ST. (**a**,**f**,**k**) Ti_65_–Zr_33_–Nb_1_–Ta_1_, (**b**,**g**,**l**) Ti_65_–Zr_31_–Nb_2_–Ta_2_, (**c**,**h**,**m**) Ti_65_–Zr_29_–Nb_3_–Ta_3_, (**d**,**i**,**n**) Ti_65_–Zr_27_–Nb_4_–Ta_4_, and (**e**,**j**,**o**) Ti_65_–Zr_25_–Nb_5_–Ta_5_.

EBSD images (inverse pole figure and phase map) of the five MEAs after ST are presented in Figure 3. The α′ and α″ phases could not be distinguished in the EBSD images because of the similarity of the crystal structures of α′ and α″. In Ti_65_–Zr_33_–Nb_1_–Ta_1_, Ti_65_–Zr_31_–Nb_2_–Ta_2_, and Ti_65_–Zr_29_–Nb_3_–Ta_3_, a large amount of feather-like α′/α″ phases and a small amount of β grains could be observed. The α′/α″ phase in Ti_65_–Zr_27_–Nb_4_–Ta_4_ and Ti_65_–Zr_25_–Nb_5_–Ta_5_ exhibited a plate-like structure. When the stability of the β phase in the alloy increased, the shape of the α′/α″ phase transformed from feather-like to plate-like [33]. Additionally, the size of the α′/α″ phase increased as the VEC of the alloy increased. The grain sizes of the residual β phase achieved through the quenching process in Ti_65_–Zr_33_–Nb_1_–Ta_1_, Ti_65_–Zr_31_–Nb_2_–Ta_2_, and Ti_65_–Zr_29_–Nb_3_–Ta_3_ were approximately 0.05 μm. Furthermore, a larger β grain size (>8 μm) could be clearly observed in Ti_65_–Zr_25_–Nb_5_–Ta_5_, which was attributable to the relatively high β volume fraction of this alloy. In addition, the α′/α″ phase precipitated at the β grain boundary and grew vertically into the β grains (Figure 3g).

### 3.2. Phase Identification

#### 3.2.1. Optical Microscopy Analysis

**Figure 4:**
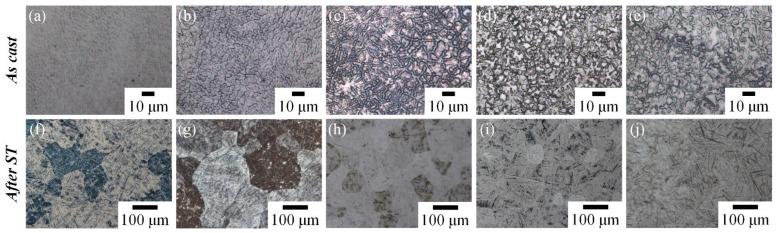
Metallographic images of the Ti–Zr–Nb–Ta MEAs in as-cast and ST states. (**a**,**f**) Ti_65_–Zr_33_–Nb_1_–Ta_1_, (**b**,**g**) Ti_65_–Zr_31_–Nb_2_–Ta_2_, (**c**,**h**) Ti_65_–Zr_29_–Nb_3_–Ta_3_, (**d**,**i**) Ti_65_–Zr_27_–Nb_4_–Ta_4_, and (**e**,**j**) Ti_65_–Zr_25_–Nb_5_–Ta_5_.

OM images of the five Ti–Zr–Nb–Ta MEAs in as-cast and ST states are presented in Figure 4. In the as-cast state, all MEAs exhibited a dendritic structure. Among the as-cast MEAs, Ti_65_–Zr_33_–Nb_1_–Ta_1_ exhibited the smallest color contrast between the dendrite and interdendrite regions, attributable to its lower melting point (<1800 °C). By contrast, Ti_65_–Zr_29_–Nb_3_–Ta_3_, Ti_65_–Zr_27_–Nb_4_–Ta_4_, and Ti_65_–Zr_25_–Nb_5_–Ta_5_ exhibited the greatest color contrast between the dendrite and interdendrite regions; this was attributable to their higher melting point (>1800 °C). The degree of compositional segregation of an alloy in the as-cast state is related to the thermodynamic parameters [6,13,29]. For example, a low δ value can facilitate the uniform diffusion of alloying elements [29]. Although Ti_65_–Zr_33_–Nb_1_–Ta_1_ had the highest δ value (4.05), its melting point was the lowest, and ΔH_mix_ was the closest to zero, which meant the lowest degree of compositional segregation in the alloy. The metallographic morphology of Ti_65_–Zr_33_–Nb_1_–Ta_1_ was still obscured by the dendritic structure because of the high δ value. In a previous study [13], as-cast Ti_65_–Zr_18_–Nb_16_–Mo_1_ exhibited both a low melting point and δ value; this indicated elemental uniformity comparable to that of the general ST state.

After ST treatment, the dendrites of the five MEAs were successfully eliminated, revealing an equiaxed and needle-like morphology. In the OM images of Ti_65_–Zr_33_–Nb_1_–Ta_1_ and Ti_65_–Zr_31_–Nb_2_–Ta_2_ in the ST state (Figure 4f,g), a large amount of needle-like structure could be clearly observed inside the equiaxed grains. In the results of EBSD, the needle-like and equiaxed grain structures were α′/α″ phase and residual β-phase grain boundaries, respectively. Furthermore, needle-like α′/α″ and numerous β-phase grain boundaries could be observed in the metallographic images of Ti_65_–Zr_25_–Nb_5_–Ta_5_ in the ST state.

#### 3.2.2. Electron Probe Microanalysis

**Figure 5:**
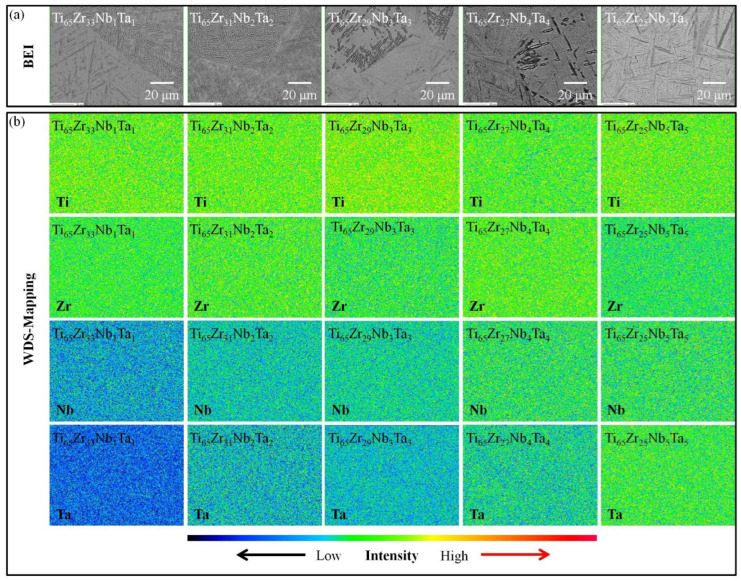
Electron probe microanalysis images of the Ti–Zr–Nb–Ta MEAs after ST: (**a**) backscattered electron imaging and (**b**) wavelength dispersive spectroscopy mapping.

The WDS–EPMA mapping images of the five MEAs in the ST state are presented in Figure 5. The Ti, Zr, Nb, and Ta in the five MEAs were distributed uniformly. Because the melting points of Ta and Nb are much higher than those of Ti and Zr, we speculated that the alloy would have a wide solidification range (T_liquidus_–T_solidus_) during solidification, which would lead to severe segregation of the alloy’s components [3]. Nguyen et al. [34] reported that Ti_25_–Zr_25_–Nb_25_–Ta_25_ must be annealed in a vacuum at 1200 °C for 24 h if a uniform composition is to be achieved. In the current study, Ti-rich MEAs with homogeneous alloy composition were obtained at a low temperature (900 °C) and within a short time (20 min). Furthermore, in the WDS mapping results, the five MEAs were not found to have considerably different compositions of α′, α″, and β phases after ST. However, the β phase of conventional Ti alloy systems contains more solute elements than the α′ or α″ phase; thus, the β phases should be easily resolvable in the mapping analysis. Because the phase structure of each alloy (β+α″+α′) in this study was not in a stable state, the compositions of the α′, α″, and β phases were not saturated, resulting in the small difference in the composition of α′, α″, and β phases. 

### 3.3. Mechanical Properties

#### 3.3.1. Bending Strength

**Figure 6:**
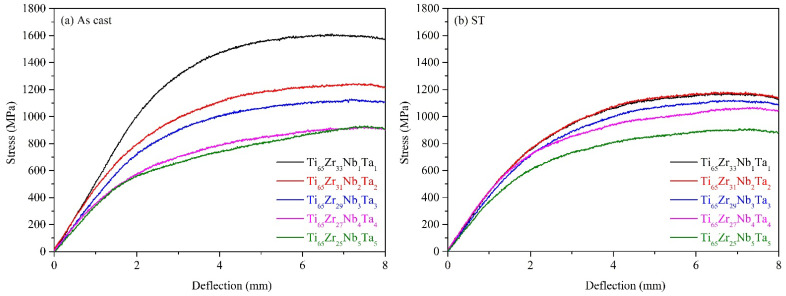
Stress–deflection curves of the Ti–Zr–Nb–Ta MEAs obtained through three-point bending tests under various conditions: (**a**) as cast and (**b**) ST.

The stress–deflection curves of the Ti–Zr–Nb–Ta MEAs in as-cast and ST states were obtained using the three-point bending test and are presented in Figure 6a,b, respectively. All MEAs reached the maximum deflection value (8 mm) in the three-point bending test, which was the same as those of Ti–6Al–4V (extra low interstitials) [29], Co–Cr–Mo [29], and 316L stainless steel [29]. The above results indicate that all MEAs had excellent deformability. Notably, double yielding was observed in the curves of both Ti_65_–Zr_27_–Nb_4_–Ta_4_ and Ti_65_–Zr_25_–Nb_5_–Ta_5_; this is characteristic of a stress-induced martensitic (SIM) transformation [35]. Because the VECs of Ti_65_–Zr_27_–Nb_4_–Ta_4_ and Ti_65_–Zr_25_–Nb_5_–Ta_5_ were close to the range 4.16–4.18 (metastable state), the SIM transformation occurred during the three-point bending test.

**Figure 7:**
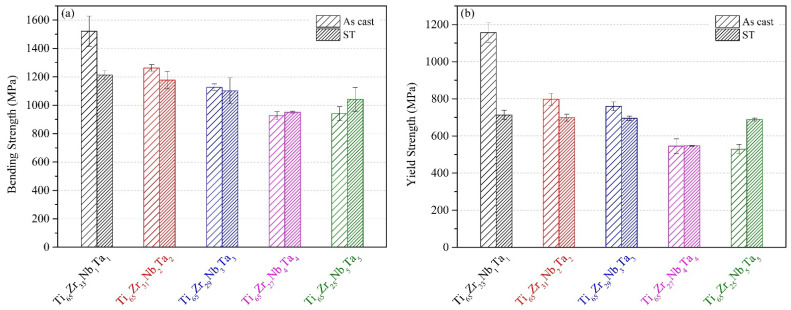
Strengths of the Ti–Zr–Nb–Ta MEAs obtained through three-point bending tests: (**a**) bending strength and (**b**) yield strength.

The bending and yield strengths of the MEAs obtained using three-point bending tests are presented in Figure 7a,b, respectively. The bending strengths of the MEAs under each condition were higher than 900 MPa, whereas the yield strengths were higher than 500 MPa. As-cast Ti_65_–Zr_33_–Nb_1_–Ta_1_ exhibited the highest bending strength (1521 MPa) and yield strength (1156 MPa). By contrast, as-cast Ti_65_–Zr_27_–Nb_4_–Ta_4_ exhibited the lowest bending strength (926 MPa), and as-cast Ti_65_–Zr_25_–Nb_5_–Ta_5_ exhibited the lowest yield strength (529 MPa). The highest strength of Ti_65_–Zr_33_–Nb_1_–Ta_1_ is mainly attributable to it having the highest δ value. However, the bending strength and yield strength of Ti_65_–Zr_33_–Nb_1_–Ta_1_ were 20% and 38% lower, respectively, after ST. As-cast Ti_65_–Zr_33_–Nb_1_–Ta_1_ exhibited abnormally high strength, which is presumed to be caused by the interaction between its high δ value and dislocation [36]. The bending strengths and yield strengths of Ti_65_–Zr_31_–Nb_2_–Ta_2_ and Ti_65_–Zr_29_–Nb_3_–Ta_3_ were lower after ST. By contrast, the bending strengths and yield strengths of Ti_65_–Zr_27_–Nb_4_–Ta_4_ and Ti_65_–Zr_25_–Nb_5_–Ta_5_ were higher after ST because the interaction between lattice distortion and the solid-solution strengthening effect was maximized after ST [37].

#### 3.3.2. Elastic Properties

**Figure 8:**
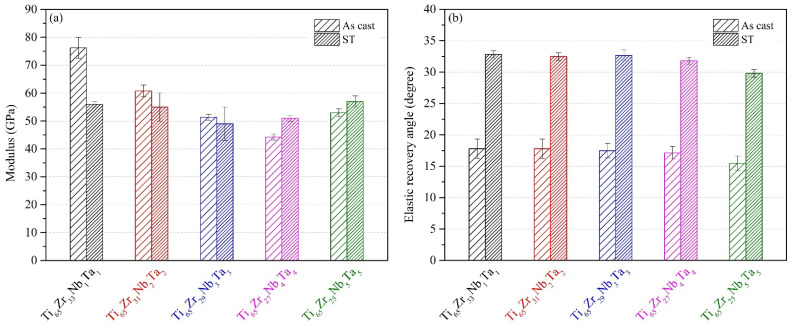
Elastic properties of the Ti–Zr–Nb–Ta MEAs obtained through three-point bending tests: (**a**) elastic modulus and (**b**) elastic recovery angle.

The elastic moduli and elastic recovery angles of the MEAs were obtained using three-point bending tests and are presented in Figure 8. The elastic moduli of the five MEAs had the same trend as their bending strength. Unsurprisingly, as-cast Ti_65_–Zr_33_–Nb_1_–Ta_1_, which had the highest bending strength and yield strength, also had the highest elastic modulus (76.2 GPa). Ti_65_–Zr_29_–Nb_3_–Ta_3_ after ST had the lowest elastic modulus (49 GPa), which was significantly lower than that of Ti_65_–Zr_18_–Nb_16_–Mo_1_ MEA (61 GPa) with metastable β phase [13]. The elastic moduli of Ti alloys are strongly related to their phase structure [38]. Studies have reported that the metastable β phase has a lower elastic modulus than the β+α″ or α″ phase [39,40], but others have found that the opposite is true [16,41]. This is because the elastic modulus of a specific phase in a Ti alloy can be highly dependent on the alloy’s composition [42,43]. Comparing the MEAs after ST, Ti_65_–Zr_29_–Nb_3_–Ta_3_ had an ultra-low elastic modulus (49.1 GPa) because of its large amount of α″ phase. A similar trend was discovered in the Ti–Nb–Sn–Zr/Fe system [41]. Both Ti–18Nb–8Sn–7Zr (wt.%) and Ti–19Nb–8Sn–0.5Fe (wt.%), which had the greatest amount of α″ phase, had low elastic moduli (~50 GPa) [41]. Conversely, Ti_65_–Zr_33_–Nb_1_–Ta_1_, which had the least amount of α′ phase, had a higher elastic modulus (56 GPa).

Ti_65_–Zr_33_–Nb_1_–Ta_1_ in as-cast and ST conditions had a large elastic recovery angle (17.8° and 32.8°, respectively), whereas Ti_65_–Zr_25_–Nb_5_–Ta_5_ in as-cast and ST conditions had a small elastic recovery angle (15.5° and 29.8°, respectively). Notably, the elastic recovery angles of the MEAs were 2-fold higher after ST because of the internal defects in the alloy being eliminated during the heat treatment. Furthermore, the elastic recovery angles of all solution-treated Ti–Zr–Nb–Ta MEAs (31.8°–32.8°) were significantly greater than those of CP–Ti (9°) and Ti–15Mo (27°) [44]. The elastic recovery ability of an alloy is strongly related to its elastic modulus, and Ti alloys with low elastic moduli generally have higher elastic recovery angles [43]. In the present study, however, Ti_65_–Zr_33_–Nb_1_–Ta_1_, which had a high elastic modulus, had the greatest elastic recovery angle. Furthermore, the elastic recovery angles of the MEAs were slightly lower than those of Ti alloys with similar elastic moduli. Both of these phenomena are attributable to the lattice distortion effect of HEAs, which hinders the elastic deformation of alloys. Because Ti_65_–Zr_33_–Nb_1_–Ta_1_ had the lowest δ value, its elastic deformability was negatively affected by lattice distortion to a smaller degree. The degrees of lattice distortion of the MEAs were much greater than those of Ti alloys, resulting in lower elastic deformation capacity. Nevertheless, all the MEAs still had small elastic moduli (49–57 GPa) and large elastic recovery angles (29.8–32.8°).

#### 3.3.3. Yield Strength–Elastic Modulus Ratios (×1000)

**Figure 9:**
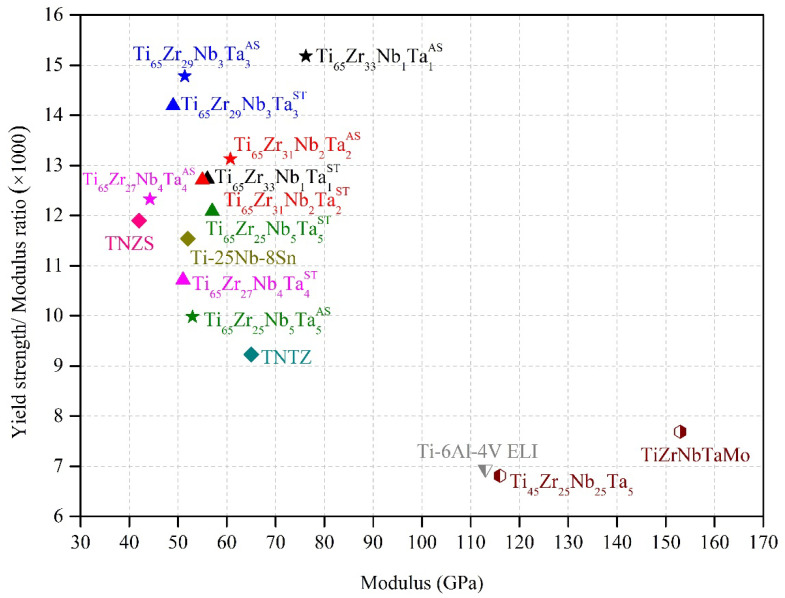
Yield strength–elastic modulus ratios (×1000) of the Ti–Zr–Nb–Ta MEAs under various conditions, several metastable β-Ti alloys [46,47,48], a bio-high-entropy alloy [3,45], and biomedical alloys [13].

The ideal biomedical implant has both high mechanical strength and a low elastic modulus. The yield strength–elastic modulus (σ_y_/E) ratios (×1000) of all MEAs in this study are presented in Figure 9. All MEAs had ultra-high σ_y_/E values (≥10) in both as-cast and ST conditions, and their values were considerably higher than those of Ti–6Al–4V extra-low interstitial (ELI) [13], biomedical HEAs and MEAs [3,45], and some metastable Ti alloys [46,47,48]. Among them, as-cast Ti_65_–Zr_33_–Nb_1_–Ta_1_ had the highest σ_y_/E value (15.2), and as-cast Ti_65_–Zr_29_–Nb_3_–Ta_3_ had the second highest σ_y_/E value (14.8). As-cast Ti_65_–Zr_33_–Nb_1_–Ta_1_ had a high σ_y_/E value because of its high yield strength (>1500 MPa), whereas as-cast Ti_65_–Zr_29_–Nb_3_–Ta_3_ had a high σ_y_/E value because of its low elastic modulus (~50 GPa). The σ_y_/E values of as-cast Ti_65_–Zr_33_–Nb_1_–Ta_1_ and Ti_65_–Zr_29_–Nb_3_–Ta_3_ were 64% and 60% higher than that of Ti–29Nb–13Ta–4.6Zr [47], respectively. Ti_65_–Zr_29_–Nb_3_–Ta_3_ also had a very high σ_y_/E value (15.2) after ST; therefore, it can potentially be used in biomedical implants.

### 3.4. Corrosion Properties

#### 3.4.1. Potentiodynamic Polarization Test

**Figure 10:**
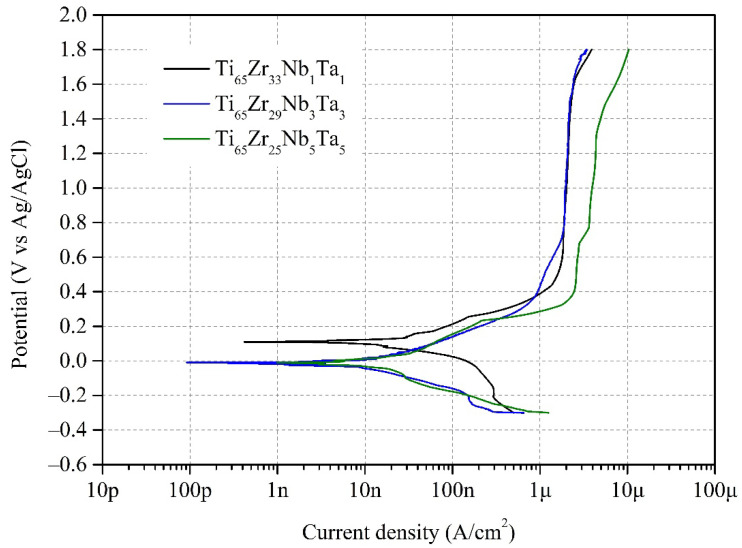
Polarization curves, obtained using potentiodynamic polarization tests, of Ti_65_–Zr_33_–Nb_1_–Ta_1_, Ti_65_–Zr_29_–Nb_3_–Ta_3_, and Ti_65_–Zr_25_–Nb_5_–Ta_5_ in artificially simulated body fluid (phosphate-buffered saline) at 37 °C.

The polarization curves of Ti_65_–Zr_33_–Nb_1_–Ta_1_, Ti_65_–Zr_29_–Nb_3_–Ta_3_, and Ti_65_–Zr_25_–Nb_5_–Ta_5_ were obtained using potentiodynamic polarization tests conducted in artificially simulated body fluid (PBS) at 37 °C and are shown in Figure 10. In addition, the corrosion potential E_corr_, corrosion current density I_corr_, passivation potential E_pass_, and passive current density I_pass_ obtained in the potentiodynamic polarization tests are presented in Table 4. All three Ti–Zr–Nb–Ta MEAs exhibited excellent corrosion resistance. Among them, Ti_65_–Zr_33_–Nb_1_–Ta_1_ has the highest E_corr_ (0.128 V), and the E_corr_ values of Ti_65_–Zr_29_–Nb_3_–Ta_3_ and Ti_65_–Zr_25_–Nb_5_–Ta_5_ (–0.026 and –0.030 V, respectively) were close to 0 V. The high E_corr_ value of Ti_65_–Zr_33_–Nb_1_–Ta_1_ may be related to its high δ value because severe lattice distortion reduces the electrical conductivity of an alloy [1], inhibiting electrochemical corrosion of the alloy. Furthermore, all three Ti–Zr–Nb–Ta MEAs exhibited very low I_corr_ values (<0.05 μA/cm^2^). The three Ti–Zr–Nb–Ta MEAs exhibited excellent pitting corrosion resistance, with their pitting potentials being higher than 1.8 V, and no pitting corrosion occurred during the test. Although the E_corr_ values of Ti_65_–Zr_29_–Nb_3_–Ta_3_ and Ti_65_–Zr_25_–Nb_5_–Ta_5_ were lower than 0 V, their E_pass_ values (0.364 V and 0.378 V, respectively) were considerably lower than that of Ti_65_–Zr_33_–Nb_1_–Ta_1_ (0.435 V). However, the I_pass_ (2.322 μA/cm^2^) of Ti_65_–Zr_25_–Nb_5_–Ta_5_ was considerably higher than those of the other MEAs. The high I_pass_ of Ti_65_–Zr_25_–Nb_5_–Ta_5_ may be related to its uniform phase composition because deleterious microgalvanic cells may form between different phase structures [48]. A passivation film rapidly forms on alloys with low E_pass_ and I_pass_ during corrosion to resist the continuation of corrosion. Therefore, Ti_65_–Zr_29_–Nb_3_–Ta_3_ exhibited the best corrosion resistance.

In Ti alloy systems, the addition of elements (such as Zr or Nb) can considerably improve the corrosion resistance of the alloy. If the Ti in the alloy investigated in this study is regarded as the solvent atom, the three Ti–Zr–Nb–Ta MEAs have the same solute atom content but exhibit clearly different corrosion resistance, which is attributable to their different ratios of solute atoms (Zr, Nb, and Ta). Different alloying elements make different degrees of contribution to the corrosion resistance of an alloy. For example, the addition of the Zr element to Ti alloys can reduce the degree of composition segregation and refine the grains such that a more uniform oxide film forms on the surface of the alloy [49]. Many scholars have proposed that the addition of Nb to Ti alloys can enhance the stability of passivation films [50]. Moreover, the occurrence of activation and depassivation can be inhibited by the addition of Nb to Ti alloys, reducing the oxide layer’s dissolution rate [51]. The effect of Ta addition on uniform corrosion and pitting corrosion resistance in Ti alloys is currently unknown, but the effect is presumed to be similar to that of Nb. Therefore, Ti_65_–Zr_29_–Nb_3_–Ta_3_ exhibited higher pitting corrosion resistance than Ti_65_–Zr_33_–Nb_1_–Ta_1_ and Ti_65_–Zr_25_–Nb_5_–Ta_5_ because of the balance among the amounts of Zr, Nb, and Ta.

#### 3.4.2. Scanning Electron Microscopy Analysis

**Figure 11:**
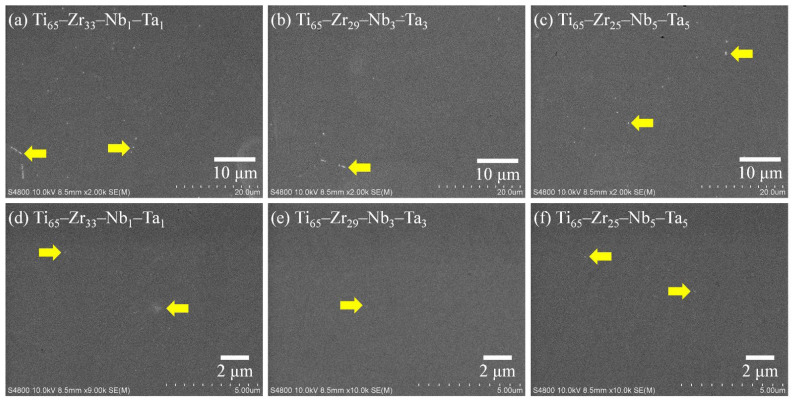
Scanning electron microscopy images of Ti_65_–Zr_33_–Nb_1_–Ta_1_, Ti_65_–Zr_29_–Nb_3_–Ta_3_, and Ti_65_–Zr_25_–Nb_5_–Ta_5_ after potentiodynamic polarization tests.

SEM images of the Ti_65_–Zr_33_–Nb_1_–Ta_1_, Ti_65_–Zr_29_–Nb_3_–Ta_3_, and Ti_65_–Zr_25_–Nb_5_–Ta_5_ alloys after the potentiodynamic polarization tests had been performed are presented in Figure 11. In the low-magnification images (Figure 11a–c), the three MEAs have a smooth surface without clear large corrosion products and pitting holes. On the surface of Ti_65_–Zr_33_–Nb_1_–Ta_1_ (Figure 11a), small white microscopic corrosion products (yellow arrow) can be observed; they are arranged in a straight line along the grain boundary. Although Ti_65_–Zr_33_–Nb_1_–Ta_1_ had a higher E_corr_, its I_corr_, E_pass_, and I_pass_ values were higher than those of the other two MEAs, resulting in more corrosion products on its surface. Conversely, a few tiny pores can be observed on the surface of Ti_65_–Zr_25_–Nb_5_–Ta_5_; these are attributable to this alloy having the highest melting point and the largest difference in melting point among alloying elements. These tiny pores were residual pores or shrinkage cavities formed during the casting process. However, the formation of a dense oxide/passivation layer on the tiny pores area of the surface is theoretically difficult, which meant that there was a preferential corrosion area. The pores may also be one of the reasons for the shift of the passivation curve of Ti_65_–Zr_25_–Nb_5_–Ta_5_ to higher values at high potentials (Figure 10). In the high-magnification images (Figure 11d–f), pitting holes cannot be observed on the surface of the three MEAs. Ti_65_–Zr_29_–Nb_3_–Ta_3_ was discovered to have small corrosion products and a void-free surface, showing that it had the highest corrosion resistance.

#### 3.4.3. X-ray Photoelectron Spectroscopy Analysis

**Figure 12:**
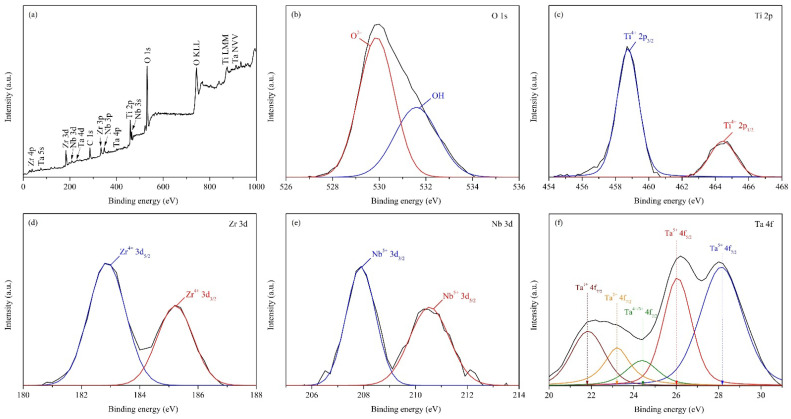
Chemical characterization of the Ti_65_–Zr_29_–Nb_3_–Ta_3_ surface after potentiodynamic polarization test: (**a**) full spectrum and (**b**–**f**) narrow scans.

The surface of Ti_65_–Zr_29_–Nb_3_–Ta_3_ after the potentiodynamic polarization test was characterized using XPS to further elucidate the corrosion behavior of the alloy. The full XPS spectrum of the Ti_65_–Zr_29_–Nb_3_–Ta_3_ surface (Figure 12a) contained peaks corresponding to Ti, Zr, Nb, Ta, C, and O. The signals of C and O were caused by surface carbon contamination and surface oxidation, respectively [52]. The narrow scans of O 1s, Ti 2p, Zr 3d, Nb 3d, and Ta 4f for Ti_65_–Zr_29_–Nb_3_–Ta_3_ are displayed in Figure 12b–f, respectively. The Ti 2p peaks in the spectrum of Ti_65_–Zr_29_–Nb_3_–Ta_3_ correspond to the Ti^4+^ oxidation state; the Zr 3d spectrum corresponds to the oxidation state of Zr^4+^; the Nb 3d peaks correspond to the Nb^5+^ oxidation state; and the Ta 4f peaks correspond to the Ta^1+^, Ta^2+^, Ta^3+^, Ta^4+^, and Ta^5+^ oxidation states. The surface oxide layer of conventional Ti-alloys often comprised metallic states (such as Nb^0^ and Ta^0^) [52], which indicates that the oxide layer is very thin and might not be sufficiently resistant to Cl^–^ ion penetration. In summary, the surface passivation layer of Ti_65_–Zr_29_–Nb_3_–Ta_3_ mainly comprised TiO_2_, ZrO_2_, Nb_2_O_5_, and Ta_2_O_5_, which meant that the alloy had excellent corrosion resistance. Furthermore, the presence of ZrO_2_, Nb_2_O_5_, and Ta_2_O_5_ in the passivation layer of Ti_65_–Zr_29_–Nb_3_–Ta_3_ enhanced the alloy’s resistance to Cl^–^ ion penetration and improved the structural integrity of the passivation film [53].

## 4. Conclusions

Five Ti-rich biomedical Ti–Zr–Nb–Ta MEAs with β+α″+α′ structure were developed by considering thermodynamic parameters and using the VEC formula. Because of the influence of the lattice distortion effects and composition segregation of the MEAs, the conventional VEC formula used for predicting phases was not entirely applicable to the Ti–Zr–Nb–Ta MEAs. Through the Ti-rich MEA design, compositional segregations in Ti–Zr–Nb–Ta were eliminated using short-time and high-temperature ST. Because of the contribution of lattice distortion effects and the three-phase structure, each Ti–Zr–Nb–Ta had an ultra-high σ_y_/E ratio. All MEA samples exhibited excellent pitting corrosion resistance; their pitting potentials were higher than 1.8 V. Ti_65_–Zr_29_–Nb_3_–Ta_3_ had excellent mechanical strength, a low elastic modulus, excellent elastic/plastic deformation ability, and excellent corrosion resistance; therefore, it can potentially be used for biomedical implants.

## Figures and Tables

**Table 1:** Energy-dispersive X-ray spectroscopy results for the Ti–Zr–Nb–Ta medium-entropy alloys (MEAs).

MEAs		Ti (at%)	Zr (at%)	Nb (at%)	Ta (at%)
Ti_65_Zr_33_Nb_1_Ta_1_	Nominal	65	33	1	1
	Actual	65.41 ± 0.13	32.14 ± 0.12	1.22 ± 0.11	1.23 ± 0.14
Ti_65_Zr_31_Nb_2_Ta_2_	Nominal	65	31	2	2
	Actual	65.62 ± 0.24	30.41 ± 0.21	2.03 ± 0.02	1.94 ± 0.04
Ti_65_Zr_29_Nb_3_Ta_3_	Nominal	65	29	3	3
	Actual	65.79 ± 0.54	28.00 ± 0.38	3.11 ± 0.12	3.11 ± 0.21
Ti_65_Zr_27_Nb_4_Ta_4_	Nominal	65	27	4	4
	Actual	65.75 ± 0.14	26.88 ± 0.31	3.95 ± 0.15	3.43 ± 0.16
Ti_65_Zr_25_Nb_5_Ta_5_	Nominal	65	25	5	5
	Actual	65.87 ± 0.39	24.34 ± 0.58	4.75 ± 0.18	5.04 ± 0.38

**Table 2:** Thermodynamic parameters of the Ti–Zr–Nb–Ta MEAs (R = 8.3145 J·K^–1^·mol^–1^).

MEAs	ΔS_mix_ (J/K·mol)	ΔH_mix_ (KJ/mol)	δ	T_L_ (°C)	Ω	VEC
Ti_65_Zr_33_Nb_1_Ta_1_	0.73R	0.17	4.05	1764	63.53	4.02
Ti_65_Zr_31_Nb_2_Ta_2_	0.80R	0.33	4.00	1782	35.94	4.04
Ti_65_Zr_29_Nb_3_Ta_3_	0.85R	0.48	3.94	1800	26.62	4.06
Ti_65_Zr_27_Nb_4_Ta_4_	0.89R	0.61	3.87	1818	21.92	4.08
Ti_65_Zr_25_Nb_5_Ta_5_	0.93R	0.74	3.79	1835	19.10	4.1

**Table 3:** A list of nomenclature for all the Greek letters and symbols used in the study.

Greek Letters and Symbols	Common Interpretation
ΔS_mix_	Mixing entropy
ΔH_mix_	Mixing enthalpy
δ	Difference in atomic radius of alloying elements
T_L_	Melting point
Ω	$\Omega =\frac{T_{L}{\Delta S}_{\mathrm{mix}}}{\left|{\Delta H}_{\mathrm{mix}}\right|}$
β	Body-centered cubic structure
α″	Orthorhombic structure
α′	Hexagonal closest packed structure
σ_y_	Yield strength

**Table 4:** Corrosion potential E_corr_, corrosion current density I_corr_, passivation potential E_pass_, and passivation current density I_pass_, obtained through potentiodynamic polarization tests, of Ti_65_–Zr_33_–Nb_1_–Ta_1_, Ti_65_–Zr_29_–Nb_3_–Ta_3_, and Ti_65_–Zr_25_–Nb_5_–Ta_5_ after solution treatment (ST).

MEAs	E_corr_ (V)	I_corr_ (μA/cm^2^)	E_pass_ (V)	I_pass_ (μA/cm^2^)
Ti_65_Zr_33_Nb_1_Ta_1_	0.128	0.0414	0.435	1.343
Ti_65_Zr_29_Nb_3_Ta_3_	−0.026	0.0116	0.364	0.856
Ti_65_Zr_25_Nb_5_Ta_5_	−0.030	0.0150	0.378	2.322

## Data Availability

Not applicable.
